# Analysis of Pathogenicity and Virulence Factors of *Ageratum leaf curl Sichuan virus*

**DOI:** 10.3389/fpls.2020.527787

**Published:** 2020-09-17

**Authors:** Pengbai Li, Chenchen Jing, Hongyan Ren, Zhou Jia, Hussein Ghanem, Gentu Wu, Mingjun Li, Ling Qing

**Affiliations:** Chongqing Key Laboratory of Plant Disease Biology, College of Plant Protection, Southwest University, Chongqing, China

**Keywords:** *Ageratum leaf curl Sichuan virus*, pathogenicity, noncognate betasatellite, C4 protein, N-myristoylation, symptom determinant****

## Abstract

*Ageratum leaf curl Sichuan virus* (ALCScV) is a novel monopartite begomovirus, which was identified from *Ageratum conyzoides* plants in Sichuan Province, China. In this study, we showed that ALCScV can induce typical dwarf and downward leaf-curling symptoms in *Ageratum conyzoides*, *Helianthus annuus*, and *Nicotiana benthamiana* plants and that the noncognate betasatellite can enhance disease symptoms and increase viral accumulation. Expression of the ALCScV-encoded V2, C1, and C4 proteins through a *Potato virus X* (PVX) vector caused severe symptoms in *N. benthamiana*. Further study revealed no symptoms in *N. benthamiana* plants inoculated with infectious ALCScV clones lacking the C4 protein and that the relative viral DNA accumulation levels significantly decreased when compared with ALCScV-inoculated plants. Thus, our mutational analyses demonstrated that C4 is a pathogenicity determinant that plays key roles in symptom formation and virus accumulation. Furthermore, we also demonstrated that the second glycine of C4 was critical for ALCScV pathogenicity.

## Introduction

Geminiviruses comprise a group of plant viruses with circular, single-stranded DNA genomes (Harrison, 1985). Based on their transmission by insect vectors, genome structures, and host ranges, geminiviruses have been classified into nine genera (*Begomovirus*, *Mastrevirus*, *Grablovirus*, *Capulavirus*, *Eragrovirus*, *Curtovirus*, *Becurtovirus*, *Topocuvirus*, and *Turncurtovirus*) (Fauquet et al., 2008; Varsani et al., 2014; Varsani et al., 2017). Among them, the *Begomovirus* genus includes the largest number of viruses and causes enormous losses to many crops plants worldwide (Fauquet et al., 2003; Navas-Castillo et al., 2011). Furthermore, depending on the genomic component, begomoviruses can be classified into those with a bipartite genome or a monopartite genome (Harrison and Robinson, 1999). Bipartite begomovirus genomes are composed of two genomic components (DNA-A and DNA-B) (Briddon et al., 2010), whereas the monopartite begomoviruses only contain one genomic component (DNA-A) (Fondong, 2013). Previous findings from some studies have shown that most monopartite begomoviruses and a few bipartite begomoviruses are associated with satellite molecules (alphasatellites or betasatellites) (Fiallo-Olive et al., 2012; Zhou, 2013). No sequence similarity has been found between satellite molecules and begomoviruses, and satellite molecules need to rely on helper viruses for replication and dissemination.

In general, for monopartite begomoviruses, the DNA A component contains six open reading frames (ORFs). ORFs V2 and V1 are contained in the sense virion strand. ORF V2 encodes the movement protein (MP), which mediates viral systemic infection of the host plant (Padidam et al., 1996), and ORF V1 encodes the coat protein (CP), which is involved in viral packaging, mobility, and vector transmission (Harrison et al., 2002). The complementary-sense strand contains four ORFs (C1, C2, C3, and C4). C1 encodes the replication-associated protein (Rep), which can initiate replication of viral DNA (Laufs et al., 1995); C2 encodes the transcriptional-activator protein (TrAP), which is primarily responsible for activating the transcription of the late viral gene (Sunter and Bisaro, 1991); C3 encodes the replication-enhancer protein (Ren), whose function is mainly to enhance viral replication in host plants (Settlage et al., 2005); and C4 encodes a multifunctional protein, which is involved in virus-induced symptom development in host plants and in systemic viral infection (Deom and Mills-Lujan, 2015; Chen et al., 2019).

Among geminivirus genomes, C4 is fully embedded in C1, although C1 and C4 use different ORFs. Recently, the C4 protein was demonstrated to act as a multifunctional protein that mainly facilitates pathogenicity, virus movement, suppressor of TGS or PTGS, and plant cell-cycle regulation (Deom and Mills-Lujan, 2015; Rosas-Diaz et al., 2018; Fondong, 2019). Recent findings revealed that the *Malvastrum yellow vein virus* (MaYVV) C4 protein suppresses RNA silencing and can enhance disease symptoms (Jing et al., 2019). The C4 protein of *Mungbean yellow mosaic virus* (MYMV) is essential for viral pathogenicity, and a C4 mutant showed significantly decreased viral DNA accumulation compared with wild-type MYMV (Sunitha et al., 2013). The C4 protein of *Cotton leaf curl Multan virus* can interact with *NbSAMS* to suppress both host plant post-transcriptional gene silencing and transcriptional gene silencing (Ismayil et al., 2018). Another study showed that the *Beet severe curly top virus* (BSCTV) C4 protein was indispensable for virus movement (Teng et al., 2010). Mei et al. showed that the *Tomato leaf curl Yunnan virus* (TLCYnV) C4 protein promoted host cell division by interacting with SKη kinase (Mei et al., 2018b).

*Ageratum leaf curl Sichuan virus* (ALCScV) is a newly discovered monopartite begomovirus that is not associated with alphasatellites or betasatellites (Li P. et al., 2018). In 2018, ALCScV was identified from *Ageratum conyzoides* plants in Sichuan Province, China. The ALCScV genome contains 2,749 nucleotides and shares the highest similarity with *Tomato leaf curl Hainan virus*. Its genome has typical geminivirus characteristics, in that it contains six ORFs distributed between the viral sense and complementary strands. In this study, an infectious clone of ALCScV was constructed that enabled systemic infection and induced typical leaf curling and dwarfing symptoms in *Ageratum conyzoides*, *Helianthus annuus*, and *Nicotiana benthamiana*. Co-inoculating ALCScV with a noncognate betasatellite induced more severe symptoms and increased viral accumulation in *N. benthamiana*. Subsequently, the molecular function of six ALCScV proteins (Rep, TrAP, Ren, CP, MP, and C4) were analyzed *via* expression with the *Potato virus X* (PVX) vector, which revealed V2 as a virulence factor that could induce systemic necrosis in *N. benthamiana*. The C1 induced severe dwarfing symptoms compared with control treatment. The C4 induced severe leaf hyperplasia and petiole elongation. Furthermore, mutational analyses showed that the C4 plays critical roles in symptom formation and virus accumulation. In addition, the second glycine (Gly) residue of C4 was indispensable for ALCScV pathogenicity.

## Materials and Methods

### Plant Materials and Growth Conditions

Wild-type *N. benthamiana*, *Ageratum conyzoides*, and *Helianthus annuus* were grown in an insect-free greenhouse, held at a constant temperature of 26°C, with a 16-h light/8-h dark cycle.

### Plasmid Construction

To test the pathogenicity of ALCScV (GenBank No. MG917698), an infectious ALCScV clone (isolate SC782) was constructed *via* Seamless Cloning (Lu, 2005). The SC782-InFu-F1/SC782-InFu-R1 primer pair (**Table S1**) was used to amplify the full-length genome of ALCScV, and this fragment was named 1.0A. In addition, the SC782-InFu-F2/SC782-InFu-R2 primer set (**Table S1**) was used to amplify a ~0.9-mer fragment (2.5 kb), which was named 0.9A. Sequencing the 1.0A and 0.9A fragments revealed an overlapping region of approximately 25 bp at both ends of the fragments. The 1.0A and 0.9A fragments were inserted into the EcoRI and SacI sites of the pBinPLUS vector to produce pBinPLUS-1.9A.

A PVX-mediated overexpression vector was constructed in order to investigate the molecular functions of six ALCScV proteins. The full-length sequences of V1 (774 bp), V2 (351 bp), C1 (1089 bp), C1_mC4_ (1089 bp) (we used the *Fast* Mutagenesis System to mutate the start codon (ATG) in the *C4* gene to ACG, without changing the amino acid sequence of the C1 protein), C2 (408 bp), C3 (405 bp), and C4 (303 bp) were amplified using specific primers (**Table S1**). The PCR products were doubly digestion with restriction enzymes and cloned into the corresponding restriction sites of the PVX vector to produce clones PVX-V1, PVX-V2, PVX-C1, PVX-C1_mC4_, PVX-C2, PVX-C3, and PVX-C4, respectively.

To construct an infectious clone of an ALCScV mutant that does not produce the C4 protein, the full-length genome of ALCScV was amplified using the primers SC782-InFu-F1 and SC782-InFu-R1. The PCR products were purified and cloned into the pGEM-T Easy vector (Promega, Madison, WI, USA). Then, we used the *Fast* Mutagenesis System (TransGen Biotech, China) and the SC782-mC4-F1/SC782-mC4-R1 primer set (**Table S1**) to mutate the first potential start codon (ATG) in the *C4* gene to ACG. In the same way, we used the SC782-mC4-F2/SC782-mC4-R2 primer set (**Table S1**) to mutate the second potential start codon (ATG) in the *C4* gene to ACG. After both potential start codons were successfully mutated, we followed the method described above to construct and infectious clone and produce pBinPLUS-1.9AmC4.

To generate the C4 mutants C4_G2A_, C4_C8A_, C4_C10A_, and C4_C8/10A_, the full-length *C4* gene sequence was amplified using the C4-F/C4-R primer set and cloned into the pGEM-T Easy vector (Promega, Madison, WI, USA). Then, we used the *Fast* Mutagenesis System and a mutation primer (**Table S1**) to mutate amino acids 2, 8, and/or 10. After DNA sequencing, the full-length sequences of C4_G2A_, C4_C8A_, C4_C10A_, and C4_C8/10A_ were inserted into the PVX vector to produce the clones PVX-C4_G2A_, PVX-C4_C8A_, PVX-C4_C10A_, and PVX-C4_C8/10A_, respectively.

To investigate the subcellular localization of C4 and C4_G2A_ proteins, the coding sequences of C4 and C4_G2A_ were amplified with the corresponding primer pairs (**Table S1**), digested with SacI and BamHI restriction enzymes and inserted into the binary vector PCHF3 to generate the recombinant vectors PCHF3-C4-GFP and PCHF3-C4_G2A_-GFP expressing the fusion proteins C4-GFP and C4_G2A_-GFP (the GFP tag was located at the Ct end of the C4/C4G2A proteins).

To further investigate the function of the second amino acid position of C4, we used the same method described above to construct an infectious clone (pBinPLUS-1.9A C4m2) that only had a mutation in the second amino acid position of C4.

### Agro-Infiltration Assays

The recombinant PVX overexpression vector and pBinPLUS-based expression vectors were transformed into *Agrobacterium tumefaciens* strain GV3101 (pMP90 pSoup). The *A. tumefaciens* bacteria were grown on LB solid medium containing rifampicin (20 μg/ml) and kanamycin (50 μg/ml) in a 28°C incubator for 48 h. Then, single clones were expanded into LB liquid medium containing rifampicin (20 μg/ml) and kanamycin (50 μg/ml) and incubated at 28°C for 12 h. The *A. tumefaciens* bacteria were collected by centrifugation and resuspended with inoculation buffer (2 mM acetosyringone, 100 mM MES, and 10 mM MgCl_2_) to and OD_600_ of 1.0. After standing at room temperature for 2 h, an aseptic syringe was used to inoculate *N. benthamiana* plants (leaf infiltration) at the 4–6 leaf stage.

### DNA Extraction and Quantitative Polymerase Chain Reaction (qPCR) Analysis

Total DNA was extracted from the newly emerged leaves using the CTAB method (Cota-Sánchez et al., 2016). A pair of quantitative primers (782-qPCR-F and 782-qPCR-R) was designed using Primer5.0 software (**Table S1**). The NovoStart SYBR qPCR SuperMix Plus Kit (Novoprotein, Shanghai, China) was used to perform the qPCR assays. qPCR was performed with 20-μl reaction mixtures containing 10-μl 2× NovoStart SYBR qPCR SuperMix Plus Kit (Novoprotein), 0.5-μl forward primer (10 μM), 0.5-μl reverse primer (10 μM), 1-μl total DNA (100 ng/μl), and 8-μl RNase-free ddH_2_O. The endogenous *Nb25S ribosomes* gene was detected as an internal control (**Table S1**). The final data were analyzed using the 2^–ΔΔCt^ method (Livak and Schmittgen, 2001). The qPCR assays were performed with three technical replicates, and at least three independent biological replicates were performed and showed similar results.

### Semi-qPCR

For semi-qPCR analysis, the quantity and purity of total DNA were measured by spectrophotometer. The total DNA concentration were normalized (100 ng/μl) prior to semi-qPCR. The quantitative primers 782-qPCR-F/782-qPCR-R were also used to semi-qPCR, and the endogenous *Nb25S ribosomes* gene was also assessed as a loading control, the semi-qPCR reaction system contains 10-μl 2× Taq Master Mix (Novoprotein, China), 1-μl forward primer (10 μM) and 1-μl reverse primer (10 μM), 1-μl total DNA (100 ng/μl), and 7-μl RNase-free ddH_2_O, and the PCR cycle number is 25. Then, the PCR products were separated by a 1% agarose gel electrophoresis.

## Results

### Pathogenicity of ALCScV

**Figure 1:**
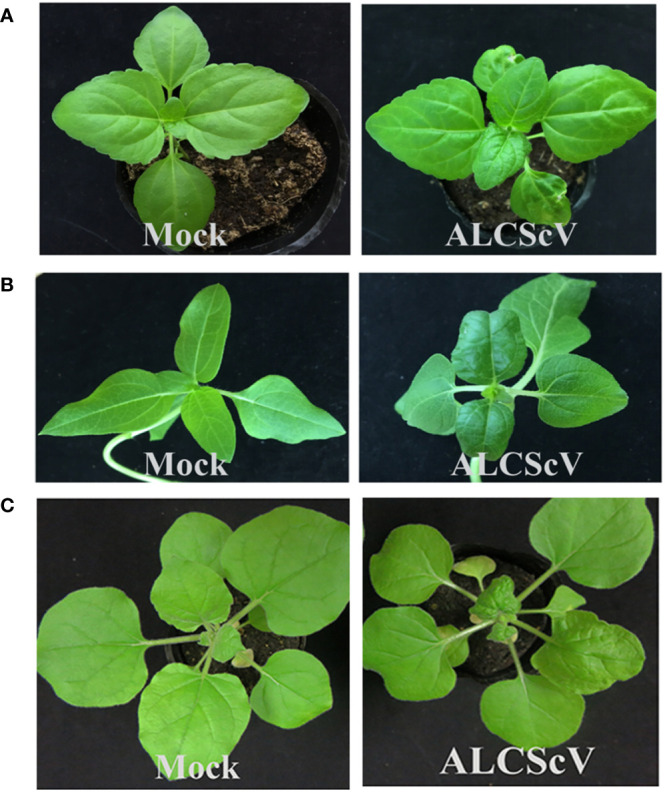
Symptoms induced by ALCScV on *Ageratum conyzoides*, *Helianthus annuus* and *N. benthamiana*. **(A**, **B)** The *Ageratum conyzoides* and *Helianthus annuus* infected with ALCScV showed leaf curling and shrinking symptoms. The pictures were taken at 14 dpi. **(C)** The *N. benthamiana* infected with ALCScV showed dwarf, enation and downward leaf curling symptoms. The pictures were taken at 14 dpi.

To investigate the pathogenicity of ALCScV, we constructed an infectious clone of ALCScV. At 14 days post-inoculation (dpi), leaf curling and shrinking started to appear on *Ageratum conyzoides*, *Helianthus annuus* and *N. benthamiana* plants inoculated with ALCScV (**Figures 1A–C**). At 21 dpi, the symptoms became more severe, with typical dwarf, enation, and downward leaf curling symptoms observed in *N. benthamiana* plants inoculated with ALCScV and no symptoms observed in mock-inoculated *N. benthamiana* plants (**Figure S1**). These results indicated the agro-inoculation with the infectious clone of ALCScV could induce typical viral symptoms on *Ageratum conyzoides*, *Helianthus annuus* and *N. benthamiana* plants.

### Noncognate DNA β Induced More Severe Symptoms and Enhanced ALCScV Accumulation

**Figure 2:**
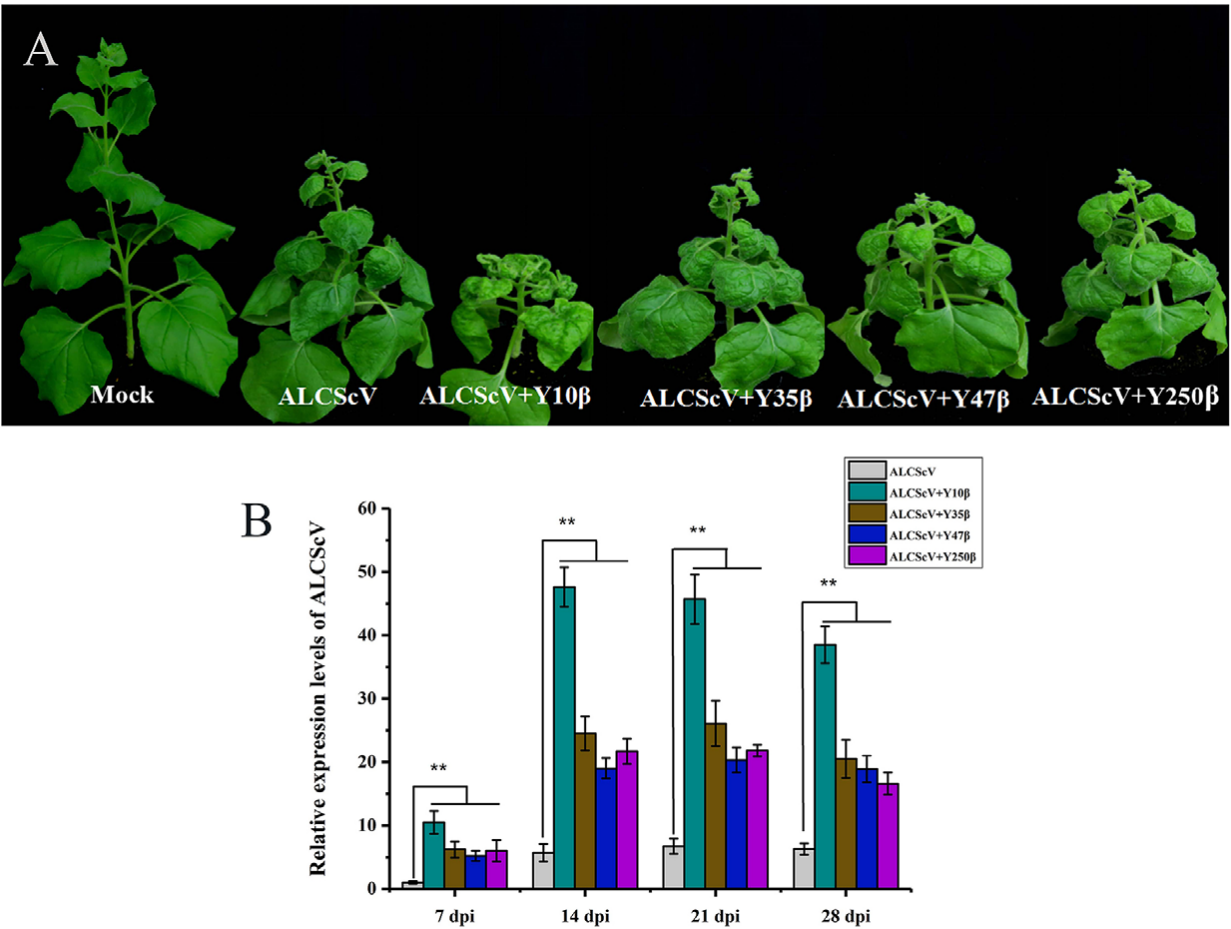
Infectivity of either ALCScV alone or together with the noncognate DNA β (Y10β, Y35β, Y47β, and Y250β) in *N. benthamiana*, respectively. **(A)** The *N. benthamiana* were photographed at 21 dpi. The results showed that the co-inoculation of ALCScV with the noncognate DNA β can enhance viral symptoms compared with the inoculation with ALCScV alone on *N. benthamiana*. **(B)** qPCR analysis of the accumulation of ALCScV in *N. benthamiana* at 7, 14, 21, and 28 dpi, respectively. The results showed that the co-inoculation of ALCScV with the noncognate DNA β can significantly increase the accumulation of ALCScV compared with the plants inoculated with ALCScV alone. “**” indicate an extremely significant difference (P < 0.01 by the Student’s t-test). The experiment contains at least three independent biological replicates.

Previous findings identified ALCScV as a new monopartite begomovirus that is not associated with alphasatellites or betasatellites, which can successfully establish systemic infection and induced typical symptoms in *N. benthamiana* plants. To investigate the effect of noncognate DNA β on ALCScV pathogenicity, we selected the *Tomato yellow leaf curl virus* (TYLCV) China betasatellite (Y10β) (GenBank No. AJ781300), MaYVV (Y47β) (GenBank No. AJ421482), *Tobacco curly shoot virus* betasatellite (Y35β) (GenBank No. AJ421484), and MaYVV Yunnan betasatellite (Y250β) (GenBank No. AJ786712) for further studies. *N. benthamiana* plants were inoculated according to the following combinations: (i) ALCScV, (ii) ALCScV + Y10β, (iii) ALCScV + Y35β, (iv) ALCScV + Y47β, and (v) ALCScV + Y250β. At 21 dpi, the results showed that co-inoculation with ALCScV and noncognate DNA β induced more severe symptoms in *N. benthamiana* plants than inoculation with ALCScV alone (**Figure 2A**). Co-inoculation with ALCScV and Y10β caused the most serious dwarfing and leaf-shrinkage symptoms (**Figure 2A**). Then, qPCR was used to determine the effect of noncognate DNA β on ALCScV accumulation, and the quantitative results showed that co-inoculation with ALCScV and noncognate DNA β significantly increased ALCScV accumulation compared with plants inoculated with ALCScV alone (**Figure 2B**). At all four time points (including 7, 14, 21, and 28 dpi), approximately 10-fold higher ALCScV accumulation was observed in *N. benthamiana* co-inoculated with ALCScV and Y10β than in *N. benthamiana* inoculated with ALCScV alone (**Figure 2B**). Meanwhile, at 28 dpi, PCR was used to determine the presence of noncognate DNA β on co-inoculation plants, and the results showed that these noncognate DNA β could be *trans*-replicated by the noncognate helper viruses ALCScV (**Figure S2**). Together, these results indicated that the noncognate DNA β can induce more severe symptoms and enhance ALCScV accumulation.

### Identification of Virulence Factors Encoded by ALCScV

**Figure 3:**
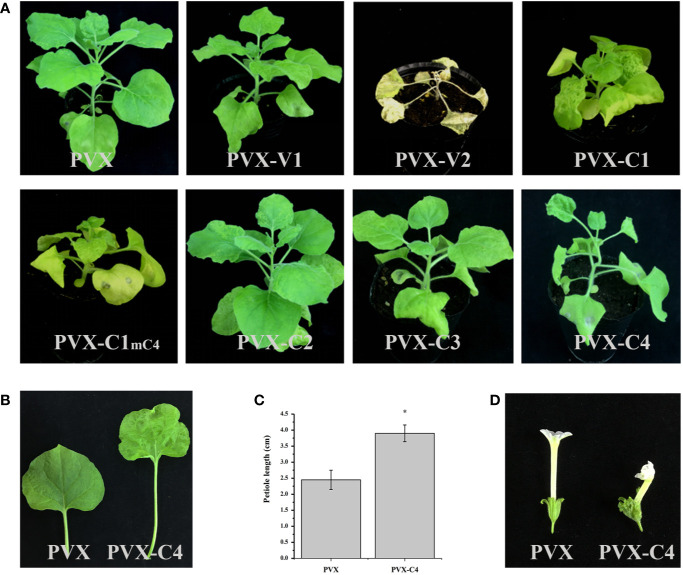
Symptoms induced by PVX-V1, PVX-V2, PVX-C1, PVX-C1_mC4_, PVX-C2, PVX-C3, and PVX-C4 on *N. benthamiana*, respectively. **(A)** PVX-V2 can induce systemic necrosis symptoms on *N. benthamiana*. PVX-C1and PVX-C1_mC4_ can induce severe dwarfing symptoms compared with control. PVX-C4 can induce severe leaf hyperplasia and petiole elongation symptoms. PVX-V1, PVX-C2, and PVX-C3 no induced obvious difference in symptoms compared with the PVX control. The *N. benthamiana* were photographed at 14 dpi. **(B)** A detailed view where PVX-C4 inoculated plants show leaf hyperplasia and petiole elongation symptoms compared with control. **(C)** The petiole length of the third leaf of the PVX and PVX-C4-infected *N. benthamiana* plants at 14 dpi. “*” indicate a significant difference (P < 0.05 by the Student’s t-test). The experiment contains at least three independent biological replicates. **(D)** PVX-C4 inoculated plants can induce flower malformation phenotype at 35 dpi.

To clarify the pathogenicity of ALCScV-encoded proteins, they were expressed in separate *N. benthamiana* plants using the PVX vector. At 14 dpi, plants inoculated with PVX-V1, PVX-C2, and PVX-C3 showed a similar phenotype (mild mosaic symptom), in contrast to plants inoculated with the empty PVX vector (**Figure 3A**). At 7 dpi, obvious leaf wilting occurred in *N. benthamiana* plants inoculated with PVX-V2 (**Figure S3**), whereas systemic necrosis was observed at 14 dpi (**Figure 3A**). Furthermore, we constructed a transient expression vector of PCHF3-V2 and inoculated with 16c *N. benthamiana*, the results indicate that the V2 protein is a suppressor of PTGS (**Figure S4**). Regarding the PVX-C1 and PVX-C1_mC4_-inoculated plants, no obvious difference in symptoms was found when compared with the PVX control at 7 dpi (**Figure S3**); however, the plants showed severe dwarfing at 14 dpi (for *C1* gene, the overlapped *C4* gene has been mutated) (**Figure 3A**). *N. benthamiana* inoculated with PVX-C4 showed leaf hyperplasia and petiole elongation at 14 dpi (**Figure 3B**). For plants inoculated with PVX-V1, PVX-C2, and PVX-C3, we also continued to observe the symptoms, at 21 and 28 dpi. They all have no typical symptoms. Statistical analysis showed that the petiole length was significantly greater in PVX-C4-inoculated plants compared to the PVX control (**Figure 3C**). With *N. benthamiana* plants in the flowering stage, the flower malformation phenotype was observed in the PVX-C4-inoculated plants (**Figure 3D**).

### Identification of ALCScV C4 as a Pathogenicity Determinant

**Figure 4:**
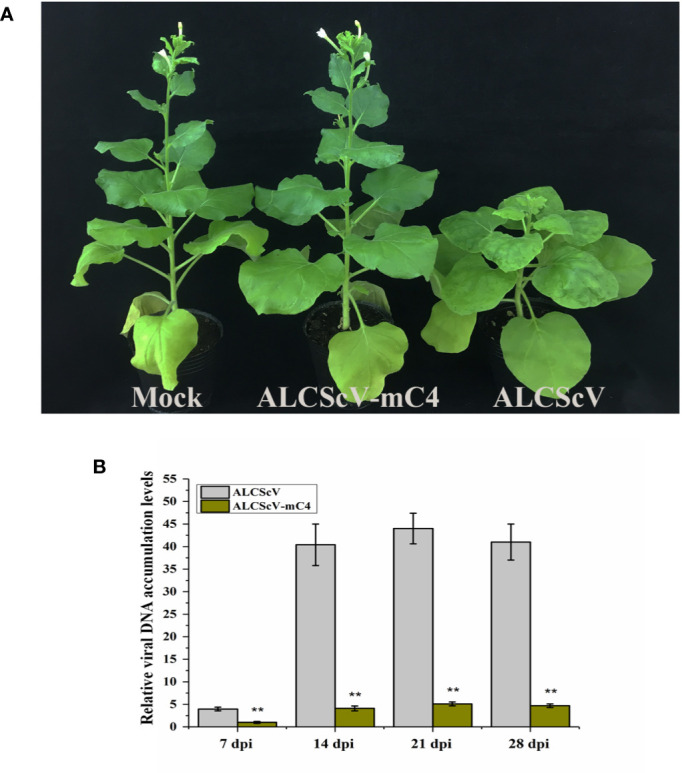
Symptoms induced by ALCScV and ALCScV-mC4 on *N. benthamiana*, respectively. **(A)** ALCScV can induce the typical dwarf enation and downward leaf curling symptoms on *N. benthamiana* at 28 dpi, ALCScV-mC4 induced no apparent symptoms on *N. benthamiana* at 28 dpi. The *N. benthamiana* were photographed at 28 dpi. **(B)** qPCR analyses the viral DNA accumulation in *N. benthamiana* inoculated with ALCScV and ALCScV-mC4, respectively. The results showed that the accumulation of viral DNA in *N. benthamiana* inoculated with ALCScV-mC4 was significantly reduced compared with the *N. benthamiana* inoculated with ALCScV. “**” indicate an extremely significant difference (P < 0.01 by the Student’s t-test). The experiment contains at least three independent biological replicates.

To further study the function of C4, we constructed an infectious clone of an ALCScV-mC4 mutant. The *C4* gene is embedded in the *C1* gene, although C1 and C4 use different reading frames. The mutation strategy employed was to mutate two potential start codons in the C4 gene (ATG, nucleotide positions 2422–2424 and 2440–2442 in the genome) to ACG, without changing the amino acid sequence of the C1 protein. Then, the *N. benthamiana* were inoculated separately with ALCScV-mC4 or ALCScV. At 28 dpi, the results showed that typical dwarf enation and downward leaf-curling symptoms were observed with *N. benthamiana* inoculated with ALCScV, whereas no symptoms were observed with *N. benthamiana* inoculated with ALCScV-mC4 (**Figure 4A**). At 28 dpi, we analyzed the integrity of the two mutated codons, finding that the mutation point was stable and that no other mutations were found (the experiment contains at least three independent biological replicates). Then, the relative viral DNA accumulation levels was analyzed by qPCR, and the results showed that relative viral DNA accumulation levels significantly decreased in *N. benthamiana* plants inoculated with ALCScV-mC4, compared with those inoculated with ALCScV (**Figure 4B**). Furthermore, semi-qPCR analysis provided similar, confirmatory results (**Figure S5**). These results suggest that C4 plays critical roles in symptom formation and virus accumulation.

### The Second Gly of C4 Was Critical for ALCScV Pathogenicity

**Figure 5:**
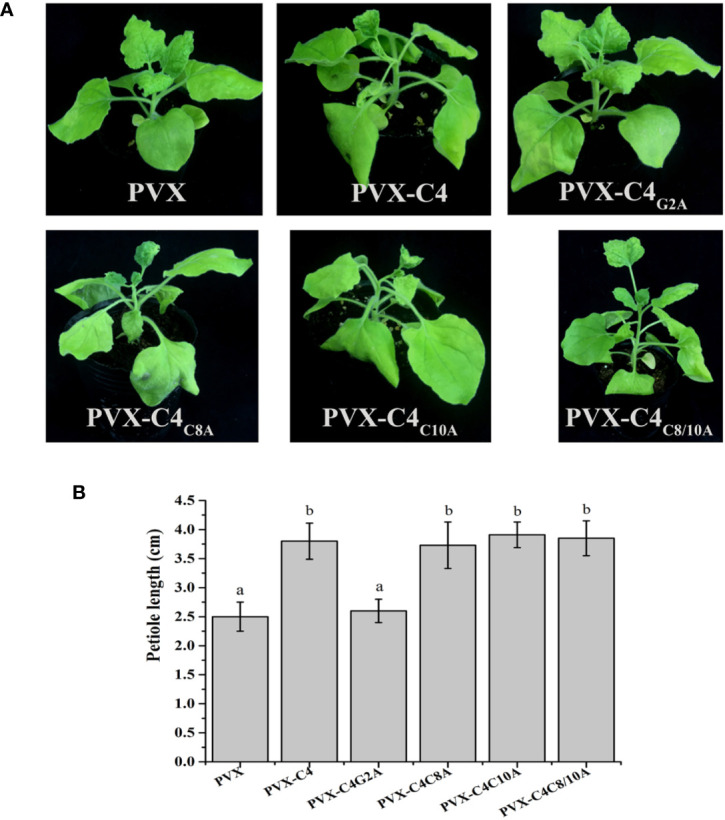
Symptoms induced by inoculations with PVX, PVX-C4, PVX-C4G2A, PVX-C4C8A, PVX-C4C10A and PVX-C4C8/10A on N. benthamiana plants, respectively. **(A)** At 7 dpi, no obvious symptoms were observed on N. benthamiana inoculated with PVX-C4G2A. The photographs were taken at 7 dpi. **(B)** Statistical analysis of the length of the 3rd petiole in the plants infected with PVX, PVX-C8A, PVX-C10A and PVX-C8/10A mutants.

**Figure 6:**
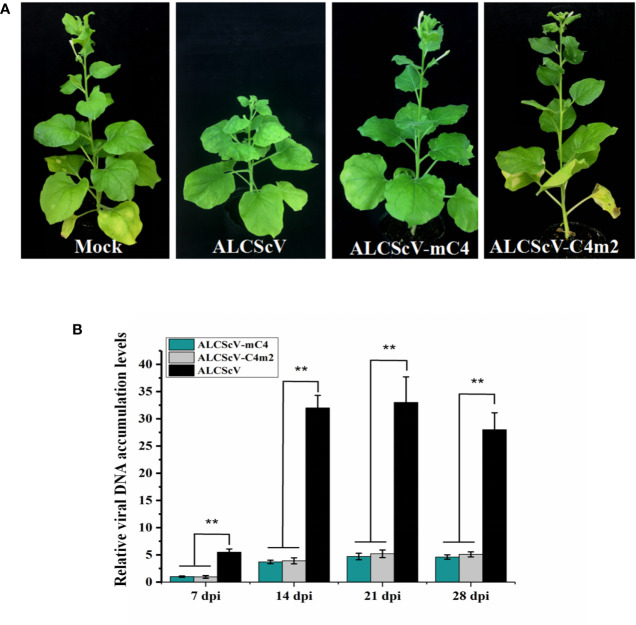
**(A)** Symptoms induced by inoculations with ALCScV, ALCScV-mC4, and ALCScV-C4m2 on *N. benthamiana* at 28 dpi. No obvious symptoms were observed in either inoculation with ALCScV-mC4 and ALCScV-C4m2 plants. **(B)** qPCR showed that the accumulation of viral DNA in ALCScV-mC4 and ALCScV-C4m2-infected plants were significantly decreased than in ALCScV-infected plants. “**” indicate an extremely significant difference (P < 0.01 by the Student’s t-test). The experiment contains at least three independent biological replicates.

Previous research showed that N-myristoylation and S-Palmitoylation are two common types of post-translational protein modification that greatly influence the functions of proteins (Stulemeijer and Joosten, 2008; Running, 2014). Thus, potential post-translational modification (N-myristoylation and S-palmitoylation) of the C4 protein was predicted using the online program, GPS-Lipid (http://lipid.biocuckoo.org/index.php). The results revealed that the C4 protein has one potential N-myristoylation site (the second Gly residue of the C4 protein) and two potential S-palmitoylation sites [the eighth and tenth cysteine (Cys) residues of the C4 protein] (**Figure S6**). The potential N-myristoylation (Gly2) and S-palmitoylation (Cys8 and Cys10) sites were individually replaced by Ala. To determine whether any of these potential post-translational modification sites affect the function of C4 protein, each C4 mutant (C4_G2A_, C4_C8A_, C4_C10A_, or C4_C8/10A_) was expressed in *N. benthamiana* from the PVX vector. At 7 dpi, the plants inoculated with PVX-C4_C8A_, PVX-C4_C10A_, and PVX-C4_C8/10A_ showed similar phenotypes, compared with plants inoculated with PVX-C4. However, the PVX-C4_G2A-_inoculated plants only showed mild mosaic symptoms, and no other typical symptoms were observed (**Figure 5A**). Meanwhile, we find same phenotypes on the length of the 3rd petiole and on flowers structure in the plants infected with PVX-C8A, PVX-C10A, and PVX-C8/10A mutants as with the PVX-C4 (**Figure 5B**). To identify whether the putative N-myristoylation site affect the localization of C4, we fused the C4 mutants with GFP obtaining C4G2A-GFP, and the result is that they are both localized in the cell membrane and nucleus (**Figure S7**). To further investigate the effect of the second amino acid site of the C4 protein on pathogenicity, we constructed an infectious clone with the potential N-myristoylation site (Gly2) in the C4 protein mutated to the Ala. At 28 dpi, the ALCScV-inoculated plants showed typical dwarf enation and downward leaf-curling symptoms, but no symptoms appeared in plants inoculated with ALCScV-C4m2 (**Figure 6A**). In addition, sequencing at 28 dpi showed that the point mutation was stable and that no other mutations occurred. Our qPCR results revealed lower relative viral DNA accumulation levels in *N. benthamiana* plants infected with ALCScV-C4m2 than in those infected with ALCScV (**Figure 6B**). Similar, confirmatory results were obtained by semi-qPCR (**Figure S8**). Taken together, these results suggest that the second Gly of C4 was indispensable for ALCScV pathogenicity.

## Discussion

In recent years, with the outbreak of *Bemisia tabaci*, begomoviruses have spread rapidly and have emerged as a major limitation of agricultural productivity throughout the world (Gilbertson et al., 2015; Jacobson et al., 2018). ALCScV is a new species of begomovirus, and its pathogenicity is unclear. In this study, we found that ALCScV can systematically infect *N. benthamiana* and induce dwarfing and downward leaf-curling symptoms. As with TYLCV, betasatellites were dispensable for ALCScV infection and the induction of typical symptoms in the host (Navot et al., 1991). Although ALCScV has only been detected in *Ageratum conyzoides* plants, the virus can be transmitted by *B. tabaci*, making it potentially harmful to agricultural production.

Previous data revealed that betasatellites can be trans-replicated by several different begomovirus, and trans-replication by a true monopartite begomovirus can potentially lead to the formation of new disease complexes in plants co-infected with other begomoviruses, thereby causing more serious symptoms (Qing and Zhou, 2009). Moreover, betasatellites play important roles in determining begomovirus host ranges and, hence, could lead to the emergence of serious disease in many crops (Jing et al., 2016; Gnanasekaran et al., 2019; Shahid et al., 2019). In this study, we found that co-inoculating ALCScV with noncognate DNA β induced more severe symptoms and increased the accumulation of ALCScV. The effect was most obvious when ALCScV was co-inoculated with Y10β. Our previous results revealed that co-inoculating *Euphorbia leaf curl virus* with Y10β induced more severe symptoms in *N. benthamiana* (Wu et al., 2011). Furthermore, previous findings showed that co-inoculating *Tobacco curly shoot virus* with the Tobacco curly shoot betasatellite can also enhance the symptoms of infection, compared to those observed after inoculation with *Tobacco curly shoot virus* alone (Li et al., 2005).

In general, the six geminivirus proteins differ in their pathogenicity. In this study, we found that over-expressing the V2 protein induced severe systemic necrosis in *N. benthamiana* plants, indicating that it functions as a key virulence factor. Many V2 proteins encoded by different geminiviruses have been demonstrated to be important virulence determinants. For example, the *Tomato leaf curl Java virus* V2 protein causes systemic necrosis by inducing hypersensitive responses in plants (Sharma and Ikegami, 2010); Working with *Apple geminivirus* (AGV), Zhan et al. observed necrotic lesions in PVX-V2 inoculated plants (Zhan et al., 2018). At 14 dpi, the PVX-C1-inoculated plants showed severe dwarfing symptoms (the overlapped *C4* gene in the *C1* gene has been mutated, so the symptom is caused only by the C1 protein). Previous research also showed that the C1 protein of *Mulberry mosaic dwarf-associated virus* can cause similar symptoms in host plants (Yang et al., 2018). Furthermore, Wezel et al. found that PVX-driven expression of C1+C4 causes local and systemic necrosis in *N. benthamiana*, and that C4 is necessary for systemic necrosis induction (using a C1_mC4_ construct) (Wezel et al., 2002). However, this phenomenon was not found in our experiments. In this study, the ALCScV C4 protein induced severe leaf hyperplasia and petiole elongation in *N. benthamiana*, and we speculate that C4 acted as a symptom determinant. Similar to our results, previous reports showed that the C4 protein from different geminiviruses acted as a symptom determinant, including the *Beet curly top virus* C4 protein, the *Sweet potato leaf curl virus* C4 protein, the BSCTV C4 protein, and the MaYVV C4 protein (Park et al., 2010; Bi et al., 2017; Li H. et al., 2018; Jing et al., 2019). Additionally, mutation analyses revealed that the ALCScV C4 protein played a key role in symptom formation and viral accumulation. No symptoms of viral infection were found in plants inoculated with *African cassava mosaic virus* harboring a C4 mutant (Hipp et al., 2016). Carluccio et al. showed that the MYMV C4 protein was indispensable for viral accumulation and symptom formation (Carluccio et al., 2018).

In organisms, post-translational protein modification is a crucial step for regulating the biological functions of proteins (Bachmair et al., 2001; Stulemeijer and Joosten, 2008). N-myristoylation and S-palmitoylation are two common types of post-translational protein modifications (Hemsley and Grierson, 2008; Traverso et al., 2008). In this study, we found that the putative S-palmitoylation site of the C4 protein did not significant affect pathogenicity. However, in some geminiviruses, S-palmitoylation of the C4 protein had different effects on its pathogenicity. Mutagenesis of the S-palmitoylation site of the AGV C4 protein reduced the symptoms of viral infection (Zhan et al., 2018). We found that inoculating plants with the PVX-C4_G2A_ variant (affecting the putative N-myristoylation site) only showed mild mosaic symptoms, and no leaf hyperplasia and petiole elongation symptoms were observed. However, further research showed that the putative N-myristoylation site of C4 protein was critical for ALCScV pathogenicity. Many previous results were consistent with our current findings. For example, the N-myristoylation site of C4 protein encoded by the *African cassava mosaic Cameroon virus* (EACMCV) was essential for membrane localization of the C4 protein, which served as the basis for EACMCV pathogenesis (Fondong et al., 2007). In the case of TLCYnV, phosphorylation and N-myristoylation of the C4 protein were critical for viral pathogenicity (Mei et al., 2018a). Meanwhile, we have also conducted mutation studies on the phosphorylation and ubiquitination of C4 protein and found that neither phosphorylation nor ubiquitination has an effect on the pathogenicity of C4 protein.

In summary, our results suggest (i) that ALCScV can successfully establish systemic infection and induce typical viral symptoms in *Ageratum conyzoides*, *Helianthus annuus*, and *N. benthamiana* plants, (ii) that the noncognate betasatellite can enhance disease symptoms and viral accumulation, (iii) that the ALCScV V2 protein can induce systemic necrosis, and (iv) that the C1 protein can induce severe dwarfing symptoms in *N. benthamiana* plants. The ALCScV C4 protein functioned as a pathogenicity determinant and the second Gly residue of C4 was indispensable for ALCScV pathogenicity.

## Data Availability Statement

The datasets generated for this study can be found in the: The GenBank accession number of *Ageratum leaf curl Sichuan virus* is MG917698.

## Author Contributions

LQ conceived and designed the experiments. PL, CJ, HR, ZJ, and HG conducted experiments. GW, ML, and PL analyzed experimental data. All authors contributed to the article and approved the submitted version.

## Funding

This research work was supported by the Fundamental Research Funds for the Central Universities (Grant No. XDJK2017A006).

## Conflict of Interest

The authors declare that the research was conducted in the absence of any commercial or financial relationships that could be construed as a potential conflict of interest.
