# Supplementary figures and images for: Analysis of Pathogenicity and Virulence Factors of Ageratum leaf curl Sichuan virus
Source: Front Plant Sci. 2020 Sep 17;11:527787. doi: 10.3389/fpls.2020.527787 (PMC7527423; doi:10.3389/fpls.2020.527787)

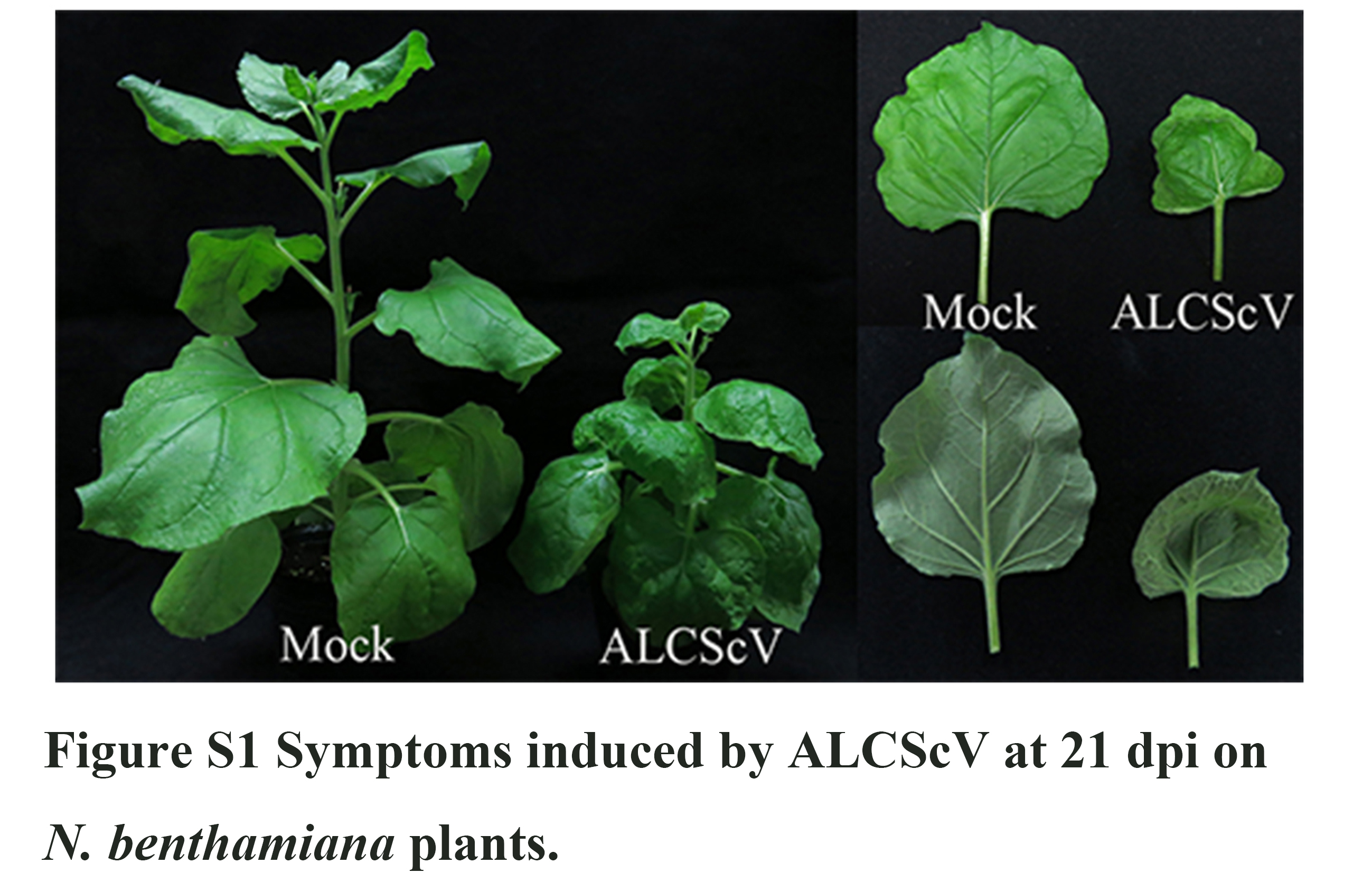

Supplement: Supplementary file 1 [file Image_1.jpeg]

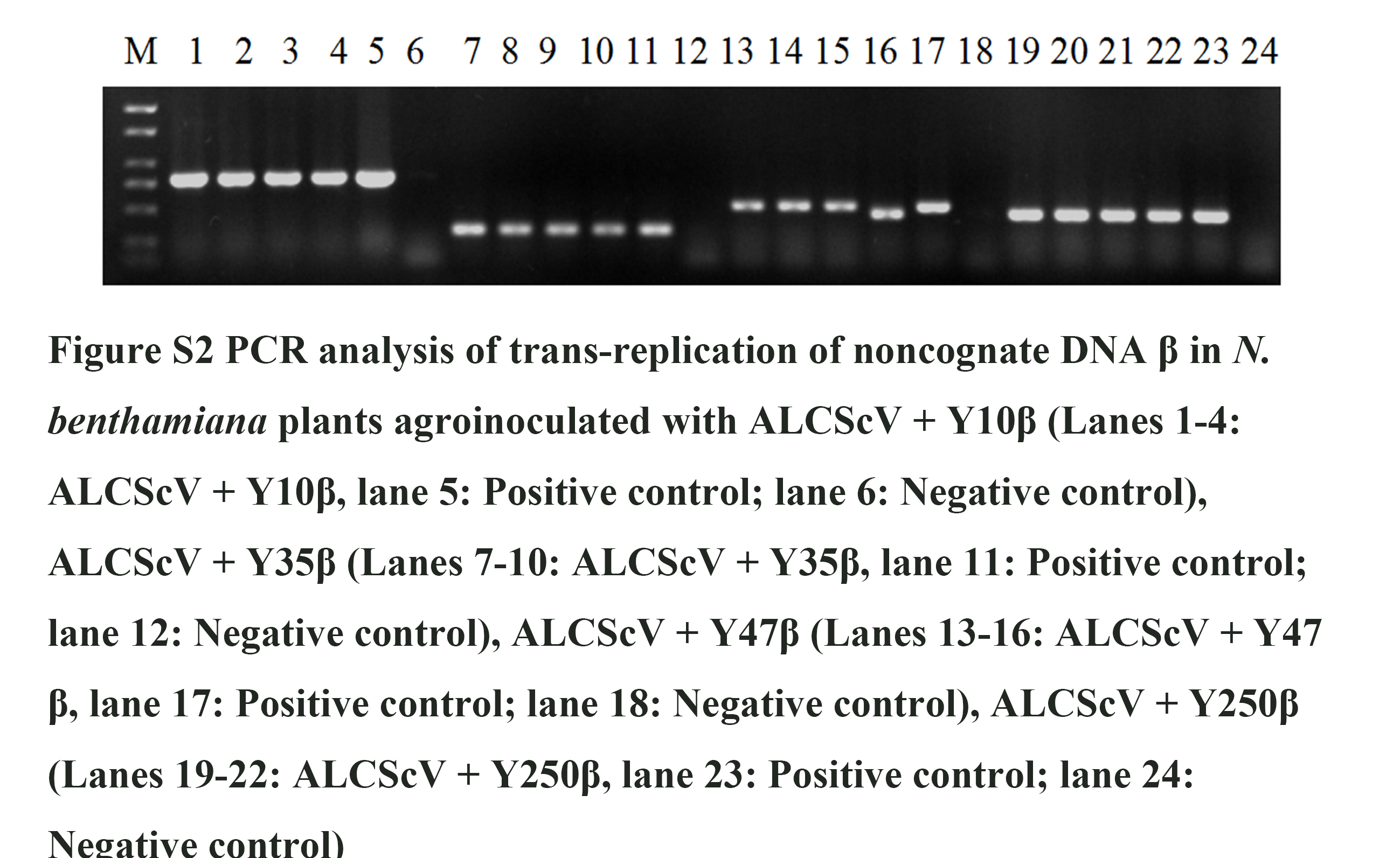

Supplement: Supplementary file 2 [file Image_2.jpeg]

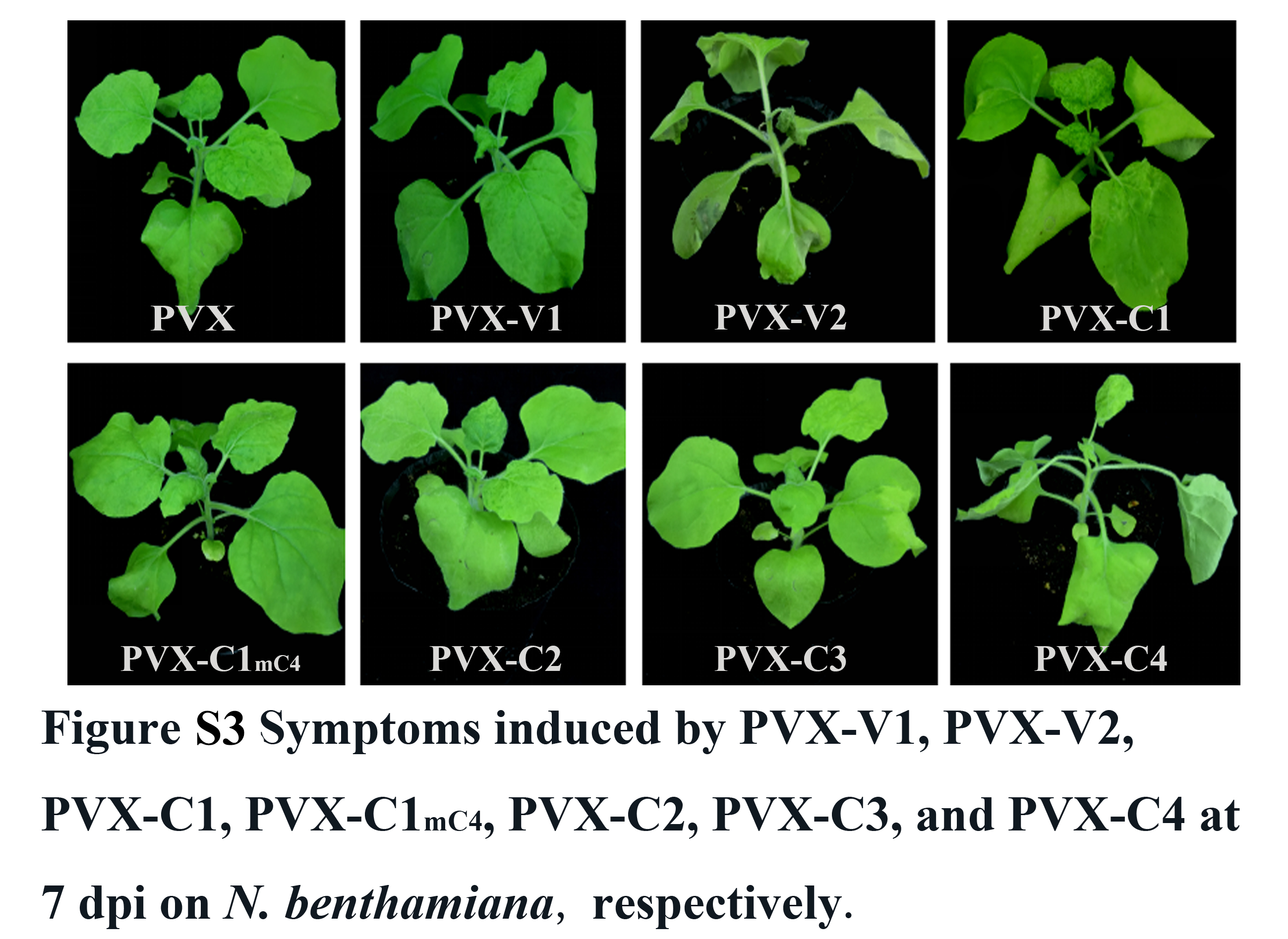

Supplement: Supplementary file 3 [file Image_3.jpeg]

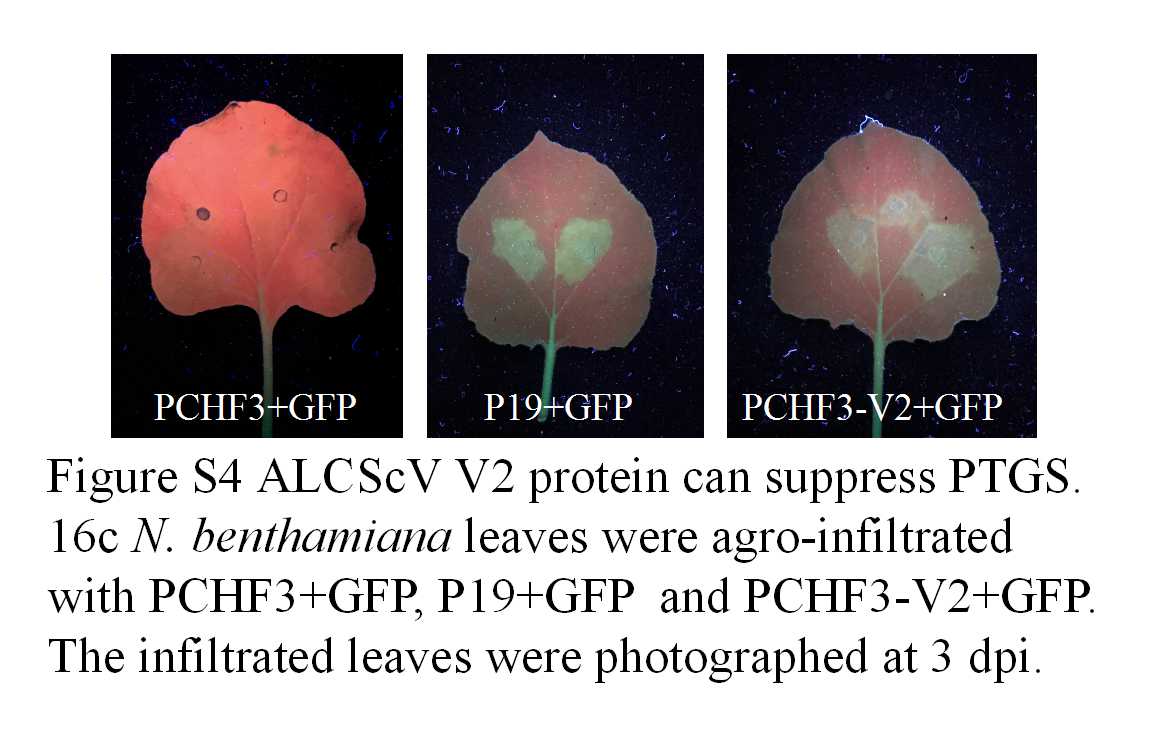

Supplement: Supplementary file 4 [file Image_4.jpeg]

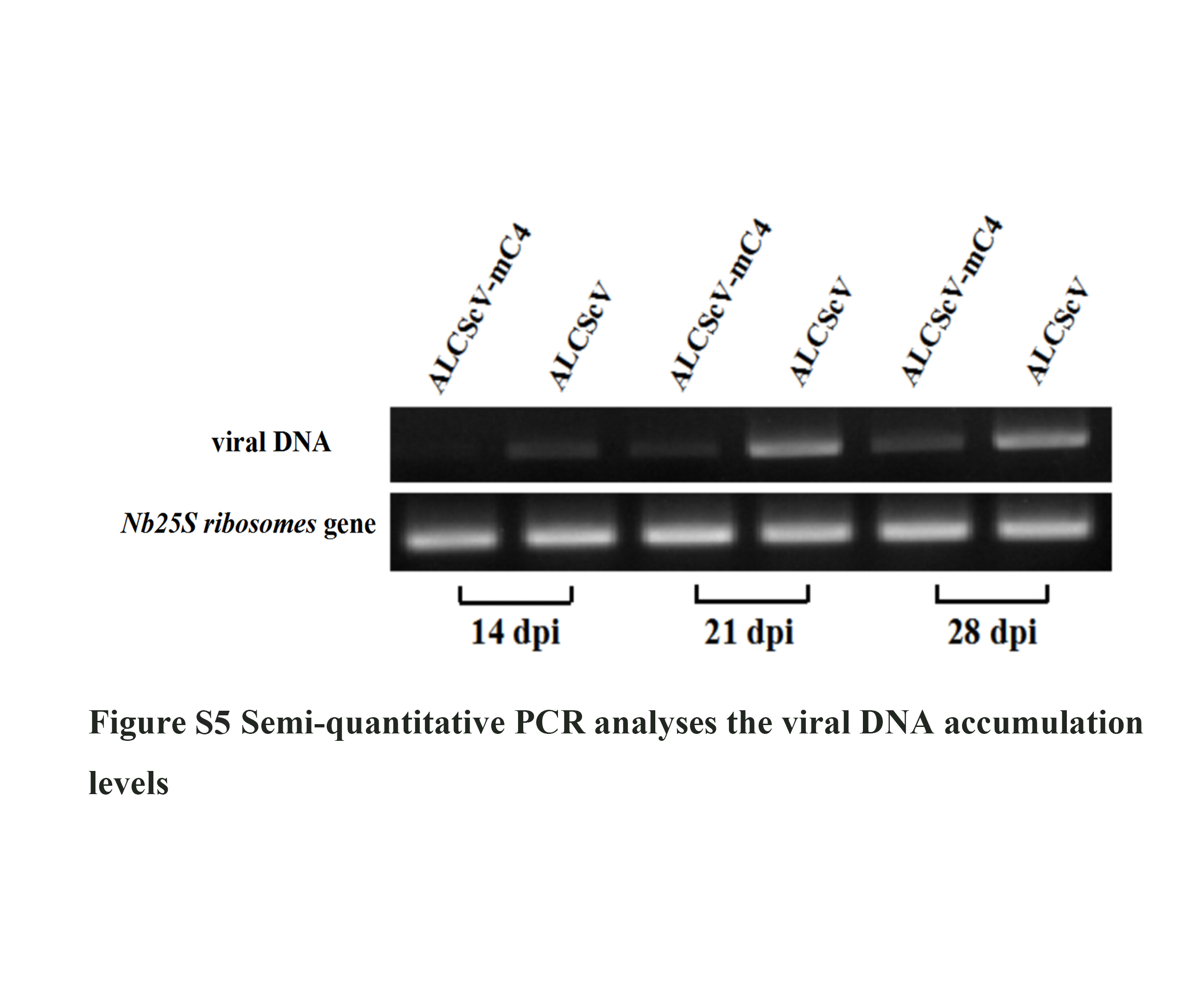

Supplement: Supplementary file 5 [file Image_5.jpeg]

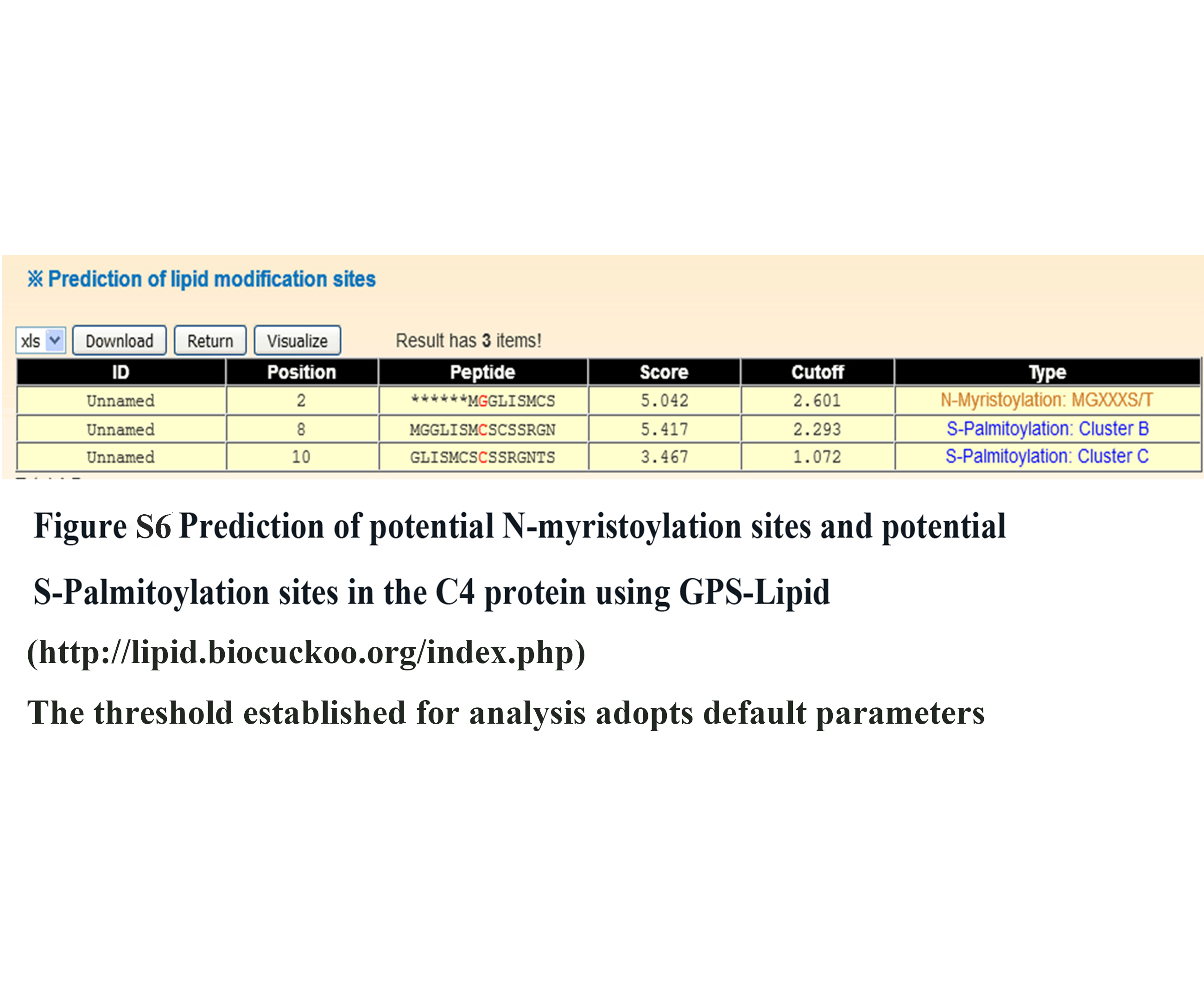

Supplement: Supplementary file 6 [file Image_6.jpeg]

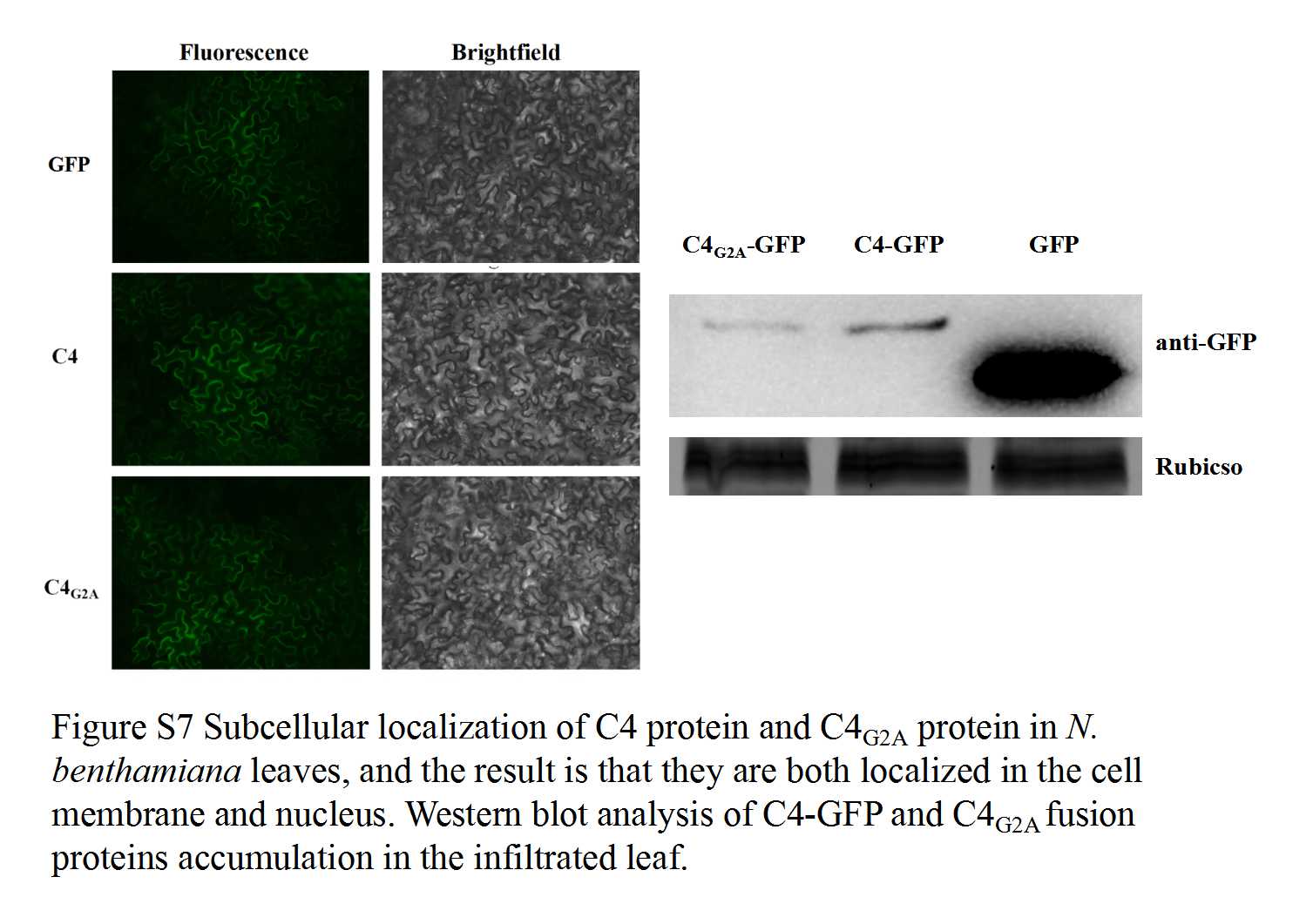

Supplement: Supplementary file 7 [file Image_7.jpeg]

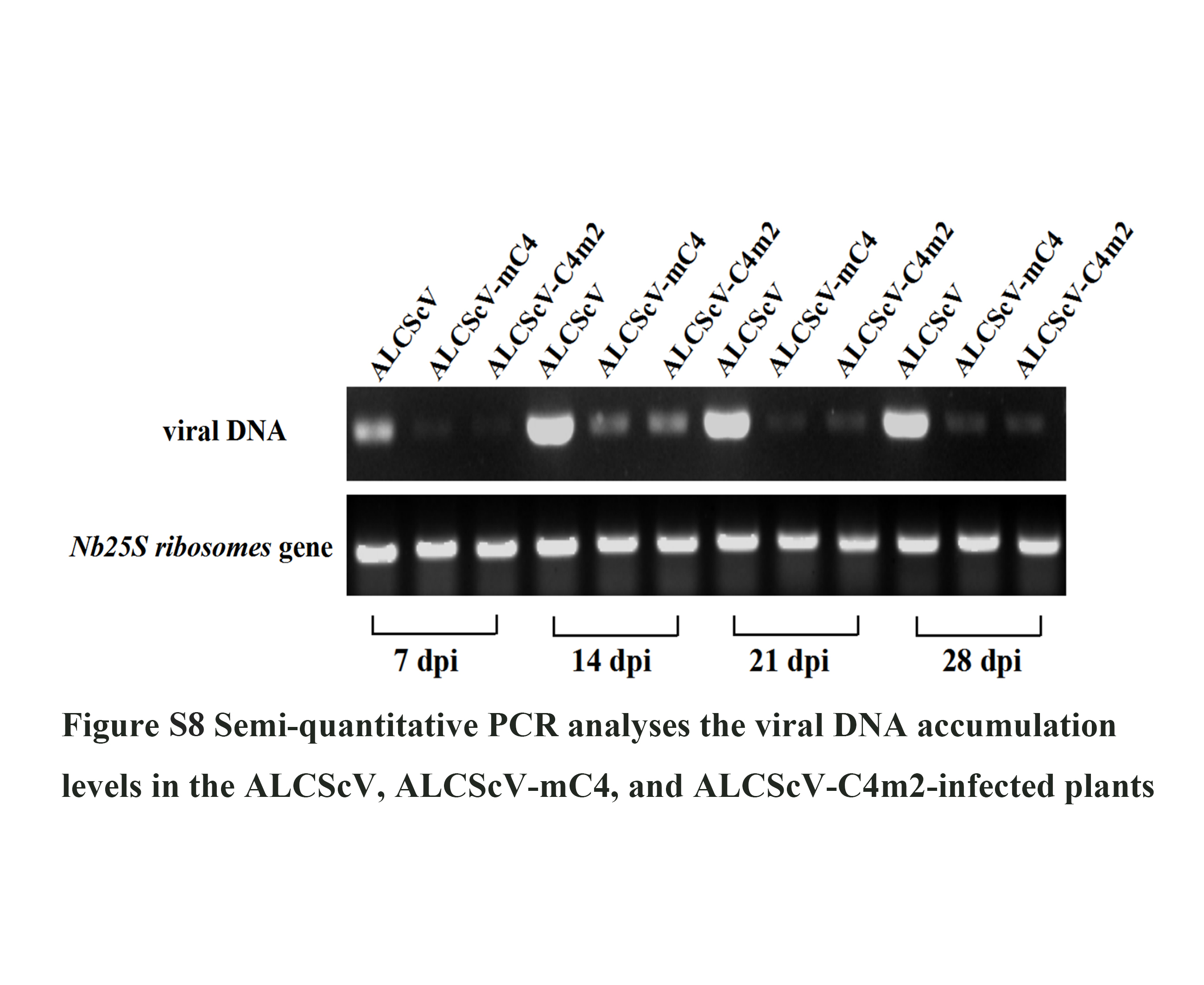

Supplement: Supplementary file 8 [file Image_8.jpeg]
