# Supplementary material for: Analysis of Pathogenicity and Virulence Factors of Ageratum leaf curl Sichuan virus
Source: Front Plant Sci. 2020 Sep 17;11:527787. doi: 10.3389/fpls.2020.527787 (PMC7527423; doi:10.3389/fpls.2020.527787)
Supplement: Supplementary file 9 [file Table_1.doc]

| Primer name | Sequence (5’-3’) |
| --- | --- |
| SC782-InFu-F1 | AAAACGACGGCCAGTGAATTGTTAATTAAGAATTCGGTACCATGTGGGATCCATTGTTAAACG |
| SC782-InFu-R1 | CAATGGATCCCACATGTTTAAAATGACTCCTTCGGGTC |
| SC782-InFu-F2 | GGAGTCATTTTAAACATGTGGGATCCATTGTTAAACG |
| SC782-InFu-R2 | CGACTCTAGAGGATCCCCGGGTACCGAGCTCAAGCTTTGAGGCGCGGTC |
| PVX-V1-F | ATCGATATGTCGAAGCGTCCC |
| PVX-V1-R | GTCGACTTAATTTGTAACGG |
| PVX-V2-F | ATCGATATGTGGGATCCATTG |
| PVX-V2-R | GTCGACTCAGGGCTTTTGAAC |
| PVX-C1-F | ATCGATATGGCTCCCTCTAA |
| PVX-C1-R | GCGGCCGCTCAACCCTCCTCCT |
| PVX-C2-F | ATCGATATGCGTACTTCGTCA |
| PVX-C2-R | GTCGACCTAAATACCCTTAAG |
| PVX-C3-F | ATCGATATGGATTCACGCACAG |
| PVX-C3-R | GTCGACTTAATAAATATTGAAT |
| PVX-C4-F | ATCGATATGGGAGGCCTCATC |
| PVX-C4-R | GTCGACCTAGTTCCCTAAGT |
| SC782-mC4-F1 | GAACTGCACGAGCACGTGGAGATGAGGC |
| SC782-mC4-R1 | CGTGCTCGTGCAGTTCGAGGGGAAATAC |
| SC782-mC4-F2 | GAGATGAGGCCTCCCGTTTTTGTGGAGC |
| SC782-mC4-R2 | CGGGAGGCCTCATCTCCACGTGCTCGT |
| PVX-C4m2-F | ATCGATATGGCAGGCCTCATC |
| PVX-C4m2-R | GTCGACCTAGTTCCCTAAGT |
| PVX-C4m8-F | CTCGAACTGCACGAGGCCATGGAGATG |
| PVX-C4m8-R | GCCTCGTGCAGTTCGAGGGGAAATACA |
| PVX-C4m10-F | TTTCCCCTCGAACTGGCCGAGCACATG |
| PVX-C4m10-R | GCCAGTTCGAGGGGAAATACAAGTGCC |
| C4-subcellular localization-F | GAGCTCATGGGAGGCCTCATCT |
| C4-subcellular localization-R | GGATCCGTTCCCTAAGTAC |
| C4m2-subcellular localization-F | GAGCTCATGGCAGGCCTCATCT |
| C4m2-subcellular localization-R | GGATCCGTTCCCTAAGTAC |
| 782-C4m2-F | ATGGAGATGAGGCCTGCCATTTTTGT |
| 782-C4m2-R | CAGGCCTCATCTCCATGTGCTCGTGCAG |
| 782-qPCR-F | CCCGTGTATGCTACTTTGAAAG |
| 782-qPCR-R | ACCTCACACCTTCAGACTGG |
| Nb25SRqF | GCGAGTAAACCCGTAAGG |
| Nb25SRqR | GCTCAGGCATAGTTCACC |

Table S1 Sequence of primers used in this study
